# Migraine and Neuroticism: A Scoping Review

**DOI:** 10.3390/bs12020030

**Published:** 2022-01-28

**Authors:** Carmen M. Galvez-Sánchez, Casandra I. Montoro Aguilar

**Affiliations:** Department of Psychology, University of Jaén, 23071 Jaén, Spain

**Keywords:** migraine, neuroticism, depression, chronic pain

## Abstract

Headache is the first cause of consultation in neurology, and one of the most frequent reasons for consultation in general medicine. Migraine is one of the most common, prevalent, and socioeconomically impactful disabling primary headache disorders. Neuroticism can be conceptualized as a disposition to suffer anxiety and emotional disorders in general. Neuroticism has been associated with various mental and physical disorders (e.g., chronic pain, depression), including migraine. With the aim to explore in depth the relationship between migraine and neuroticism, and contribute to the understanding of this relation in order to provide a better treatment for migraine patients based on a personalized and more comprehensive approach, a scoping review was performed using PubMed, Scopus, and Web of Science. Databases were searched independently by the two researchers, reaching a final set of 18 articles to be included. The search terms were: migraine and neuroticism. Neuroticism seems to be highly prevalent in migraine patients. Findings reveal that migraine patients with comorbid depression and anxiety showed higher levels of neuroticism. Depression has been associated with an increased risk of transformation from episodic to chronic migraine whereas neuroticism might be a mediator factor. Neuroticism also might be a mediator factor between childhood maltreatment and migraine. The revision conducted confirms that: (1) Migraine patients usually have a higher level of neuroticism and vulnerability to negative affect, compared to non-migraineurs and tension-type headache patients. (2) Neuroticism is associated with migraine. Nonetheless, more research is needed to clarify potential moderators of this relationship and the role of neuroticism itself in this disease. This knowledge might be useful in order to promote a better management of negative emotions as part of intervention programs in migraine.

## 1. Introduction

Headache is the first cause of consultation in neurology, and one of the most frequent reasons for consultation in a general medicine office [1]. They are classified as primary when there is no organic or other known reason, and secondary when there is an organic sign. Among the primary headaches are migraine, tension-type headache, cluster headache (acuminate, histamine, or Horton), and others not classified within the aforementioned types [2,3]. Although tension-type headache seems to be the most frequent in daily practice, it is no less true that migraine is precisely the most disabling from the social, economic, and psychological points of view [1,2,3,4].

In effect, migraine is one of the most common disabling primary headache disorders and several epidemiological studies have confirmed its high prevalence and negative socio-economic and personal impact (i.e., problems at work, in relationships, in academic field, in social spheres, etc.) [5]. In the 2010 Global Burden of Disease Study (GBD), migraine was ranked as the third most prevalent disorder in the world. Some years later, in the 2015 GBD, it was the third highest cause of disability worldwide in both males and females under the age of 50 years [5]. The evident burden of headaches provoked a call to action by the Word Health Organization (WHO) in different years (i.e., 2004, 2011). On purpose, WHO, in association with the non-governmental organization Lifting the Burden, participates in the Global Headache Campaign. This initiative, launched in 2004 and still active today, aims to raise awareness of the problem and also improve the quality of care and access to it provided to people with headaches worldwide [6].

In addition, personality remains as a central aspect in psychology and therapy. One possible explanation factor is the need to consider the personality traits (i.e., habitual patterns of behavior, thought, and emotion such as neuroticism, psychoticism, extraversion, coping strategies, etc.) in the treatment of each disease [7,8]. By including personality traits in treatment and management of illnesses, it would make possible a more personalized approach for these patients and it seems to be related to a reduction of medication overuse [8]. In this sense, neuroticism is one of the most studied personality traits due to is implications for the health, not only mental but also physical [8,9]. Neuroticism can be considered as a disposition to suffer anxiety and emotional disorders in general [10]. Moreover, neuroticism has been associated with various mental disorders (especially anxiety and depression), the morbidity and mortality of various physical diseases (including migraine, fibromyalgia, rheumatoid arthritis, temporomandibular disorder), a greater number of medical complications, increasing frequency of health services use, and worse prognosis and health-related quality of life [9,11,12,13,14]. Moreover, it is important to notice that migraine patients used to show higher level of neuroticism and vulnerability to experience negative affect, compared to non-migraineurs patients [15,16,17,18,19,20,21].

Based on previous described literature, the current scoping review has as its main objective to explore in depth the relationship between migraine and neuroticism. This revision aims to contribute to the understanding of this relation in order to provide a better treatment for migraine patients based on a personalized and more comprehensive approach.

### Migraine

**Figure 1:**
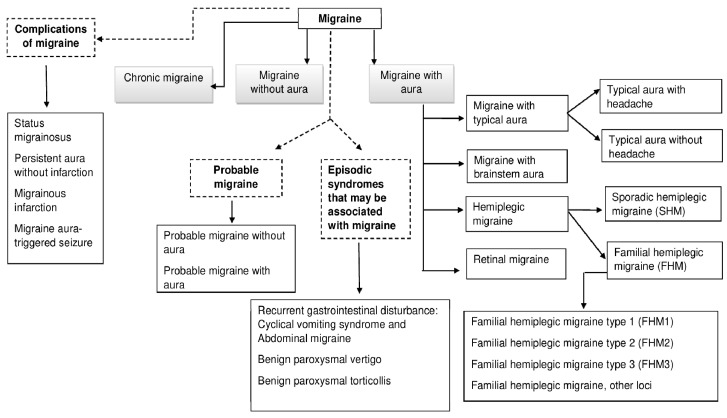
Classification of Migraine.

Based on the Headache Classification Committee of the International Headache Society (IHS), migraine can be conceptualized as a neurological disorder that manifests itself in a severe headache [5]. Migraine includes two major types. On one hand, there is migraine without aura, which is a clinical syndrome characterized by headache with specific features and associated symptoms [5]. On the other hand, there is migraine with aura, which is primarily characterized by the transient focal neurological symptoms that usually precede or sometimes accompany the headache [5]. The name of each type of migraine comes from the fact that some migraines are preceded by mostly visual sensations called auras (warning), while others are not, that is, they occur without aura [1]. In the same line, the common migraine is at least three times more common than migraine with aura [1]. Additionally, some patients also experience a prodromal phase, which takes place hours or days before the headache, and/or a postdromal phase following headache relief. Prodromal and postdromal symptoms comprise hyperactivity, hypoactivity, depression, cravings for particular foods, fatigue, and neck stiffness and/or pain (which might become chronic), among others [5]. Figure 1 shows the detailed types of migraine elaborated by authors according to HIS [5].

The pathophysiological ethology of migraine is still under research. However, one of the main conceptions states that migraine is a neurovascular disorder associated with cortical hyperexcitability [22]. Although the circumstances that trigger chronic migraines are unknown, there is electrophysiological and neuroimaging revealing that morphological, functional, and chemical changes occur in the central nervous system [22,23].

In the case of a migraine with aura, the disseminated neuronal depression or depolarization is an electrophysiological phenomenon, which seems to be responsible for triggering the headache attacks [24]. It consists of a depolarization of the membrane of neurons and glial cells in the cortex, which spreads through the rest of the brain areas at a speed of approximately 3 m/min. After an intense initial depolarization with a massive efflux of K^+^ and glutamate to the extracellular environment, there is hyperpolarization of the adjacent neurons, which facilitates the spread of neuronal depression. Functional neuroimaging has shown that, during a crisis, there is a transitory change in oxygen at the brain level consistent with the retinal topography of the aura. Moreover, disseminated cortical depression is able to activate the central and peripheral trigeminal-vascular nociceptive pathways of pain [24].

The sensitization phenomena of the trigeminal-vascular pathway are also clinically evident during migraine attacks. The sensitization of the first-order neuron of this pathway results in the characteristics of pulsatile, unilateral pain, which is aggravated by exertion. When the second-order neuron (central, located in the spinal trigeminal nucleus) is sensitized, the result is scalp hypersensitivity or cutaneous allodynia. Finally, extracephalic allodynia (in the trunk and/or upplimb) occurs when the third-order neuron (thalamus) is sensitized. Sensitization phenomena are more common in people with chronic migraine, and seem to be present in a higher percentage when the migraine attacks are more frequent. It has been suggested that it is due to sensitization of the central nociceptive pathway. This alteration, together with a lower threshold for pain and abnormal processing of nociceptive impulses, is the pathophysiological basis of the chronification of migraine. The progressive dysfunction of the central nociceptive system as a consequence of the repetition of migraine attacks could be a mechanism by which migraine becomes a chronic disorder [23,25].

Different studies have reported autonomic abnormalities associated with migraine. It has been reported that a decrease or absence of reciprocal inhibition between the two nuclei coeruleus exists, which probably leads to increased parasympathetic activity in people with bilateral migraine, while on the contrary, the reciprocal inhibitory activity would decrease activity parasympathetic in a unilateral migraine [26]. Asymmetry in blood flow velocity brain secondary to persistent vasomotor changes has also been described, and is consistent with the asymmetric distribution of perfusion and metabolism associated with cortical disseminated depression [27]. Otherwise, brain self-regulation during headache in migraineurs is different, and is even similar to total autonomic block experimental blood vessels [28]. All these findings might be in relation to a possible loss of the sympathetic and parasympathetic control of cerebral blood flow [28,29].

Metabolic abnormalities have also been demonstrated through multiple studies. On purpose, it has been found that there is increased lactate in cerebrospinal fluid, cyclic adenosine monophosphate (including 48 h after the episode), decreased magnesium, increased neuronal glucose consumption, decreased activity of NAHD-dehydrogenase, citrate synthase, and cytochrome oxidase c, suggesting an alteration of mitochondrial oxidation. The decreased secretion of platelet and circulating adenosine triphosphate (ATP), probably secondary to abnormalities of purinergic metabolism, has been further reported. Similarly, a decrease in phosphocreatine and an increase in inorganic phosphate, reversible decrease of 10 to 15% of acetylaspartate (especially in migraine with aura), a decrease of intracellular magnesium (20%) proportional to the degree of brain bioenergetic dysfunction, a decrease in mitochondrial ATP production, and reduction in cytosol acidification that suggests a decrease in glycolytic efflux have been observed. Finally, the neuronal phosphodiesterase decreases of phospholipid instability in the membrane neuronal have been proposed as a contributor for the hyperexcitability phenomenon observed in migraine [28].

Apart from this, migraine has a strong hereditary component. In fact, more than 50% of patients have a clear family history of migraine. In recent years, great efforts have been made trying to unravel the genetic substrate of migraine. Thus, three genes have been identified for one variant, remarkably rare, autosomal-dominant migraine: the Familial hemiplegic migraine [3]. The genetic basis of migraine and neuroticism needs to be studied in depth, especially for its implications in the pharmacological treatment. At this regard, abortive or symptomatic treatment is mandatory in all migraineurs [2,3]. Medications for the treatment of migraine attacks can be divided into nonspecific, specific, and adjuvant [2,3]. Medications for Non-specific include pain relievers and Non-steroidal anti-inflammatory drugs (NSAIDs) [2,3]. The specific ones include ergotics and agonists of the 5-HT1B/D receptors, commonly known as “Triptans” (i.e., Sumatriptan, Zolmitriptan, Naratriptan, Rizatriptan, Almotriptan, Eletriptan, and Frovatriptan) [3]. The adjunctive medications are mainly antiemetics/prokinetics (i.e., domperidone, metoclopramide), necessary in patients with nausea and vomiting [3]. Analgesics are of very little use in the treatment of migraine headaches [2,3]. Is highly recommended to avoid painkiller combinations with barbiturates, codeine, and/or caffeine, due to the risk of developing chronic daily headache due to the abuse of these drugs [2,3].

In addition, regarding the treatment of migraine, it is recommended to rule out intracranial disease before following warning signs: abrupt onset of symptoms, first appearance after 55 years of age, occipitocervical location of pain, or presence of abnormal neurological signs [30]. A headache that increases rapidly in frequency or the appearance of pain that awakens the patient in the early morning seems to be also related with a poor prognosis [30].

The management of migraine currently requires multidisciplinary support, especially when it is of high frequency or is chronic, considering multiple pharmacological and non-pharmacological aspects. Three main areas of management should be considered: management of comorbidities, non-pharmacological management, and pharmacological management—acute and preventive [2].

Symptomatic treatment is indicated when migraine attacks occur less than 4 days per month and are not disabling [31]. Gradual treatment is preferred, starting with the drug that is appropriate for the severity of pain (intensity and duration) and depending on the patient’s previous response to drugs, either those specific for migraine or anti-inflammatory medication. The selected agent should be used at the lowest effective dose and as early as possible in relation to the onset of pain. It is better to choose products with only one active agent and to continue with rescue medication if the first-line drug does not end the crisis [1,32].

The goal of preventive therapy is to reduce the frequency and severity of attacks by at least 50%, reduce dependence on acute medication, and improve the health-related quality of life of people with migraine headaches [32]. In the same line, it is recommended to: (1) start with the minimum dose of preventive medication and increase it if necessary until the effective dose is reached, as well as (2) to start with monotherapy, (3) maintain the treatment for at least three months, (4) to inform the migraine patient about the several weeks requirement for an apparent effect (while rescue medication may be indicated), (5) to minimize side effects and improve compliance, (6) to choose the drug according to the present comorbidity and potential drug interactions, and (7) to consider the adverse events associated with each agent. The currently most favored therapeutic approach is to target the pathophysiological mechanisms of migraine [32].

The prevention and treatment of migraine also implies some general lifestyle modification measures, such as: cutting out caffeine, getting regular physical activity, and eating and sleeping on a regular schedule. Moreover, psychiatric comorbidity (especially anxiety and depression) should also be treated [33]. In addition, triggers or aggravating pain elements, medication abuse, and the use of combined analgesics and opiates should be avoided.

Likewise, false expectations should not be created such as claiming the total disappearance of the attacks or the complete cure of the disease. Migraine is a clinical condition that usually persists for the majority of life and in most of the cases, a 70% decrease in the intensity and frequency of the attacks is considered a satisfactory goal [30]. Patient education is also another essential point for the successful treatment of migraine. In order to know the nature of the disease and increase the effectiveness of the treatment, the individual must look for and avoid possible triggers (i.e., stress, some foods and drinks, hormonal changes in women, sensory stimuli such as bright lights and loud sounds, insomnia and hypersomnia, intense physical exercise, marked changes in temperature, some medications, etc.) [1].

## 2. Methodology

**Figure 2:**
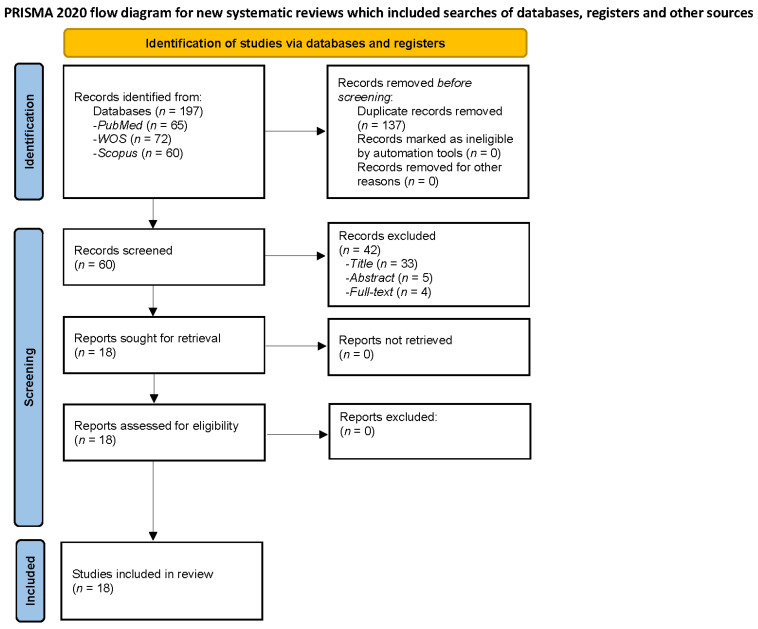
Flow diagram of Migraine and Neuroticism.

PubMed, Scopus, and Web of Science databases were searched independently by the two researchers. Discrepancies were resolved by consensus. The authors independently screened all articles and selected those that satisfied the inclusion criteria (see next paragraph for a better comprehension) for full text analysis. The titles and abstracts of the articles were screened to remove irrelevant studies; the remaining shortlisted articles were analyzed in depth for eligibility. The full texts of relevant articles were retrieved and screened, following the inclusion and exclusion criteria, to reach a final set of 18 articles to be reviewed. The screening and selection for the inclusion processes are shown as a PRISMA flowchart (Figure 2). The last search was conducted on 20 November 2021. The search terms were as follows: migraine and neuroticism.

Eligibility criteria: Studies were included if they (1) were peer-reviewed original studies, (2) included migraine patients, and (3) were focus on migraine and neuroticism. The exclusion criteria were as follows: (1) review article or meta-analysis; (2) comment, editorial, case report, letter, or meeting/congress abstract; and (3) articles written in another language different from Spanish and English.

## 3. Results

### 3.1. Neuroticism Review’s Findings

Results of the review confirm that, nowadays, personality continues being a central theme in psychology and therapy due to the relevance of personality traits in the design and implementation of more effective and personalized treatments [8]. There is no theory of human behavior that does not address this question or include it in some way as trait, individual difference, or related to the self-concept, self-esteem, etc.

Concerning the study of personality, in recent years, several authors have defended a perspective more dimensional compared to the classic categorical. This perspective falls on dimensional models such as the big five model, and propose that neuroticism is the trait of personality more consistently associated with psychopathology [11]. However, other authors consider that some pathological dimensions of personality are not adequately represented in existing dimensional models (i.e., dependency or psychoticism) [34]. Therefore, it would be convenient to have an evaluation that integrates both models [34], further considering the study of the traits of pathological personality traditionally included in categorical models and its association with mental health.

Neuroticism is a dimension related to the disposition to suffer what classically is known as neurotic disorders, including both anxiety and emotional disorders in general. An individual with high neuroticism used to be anxious, depressed, tense, irrational, shy, sad, emotional unstable, and distinguished by low self-esteem and frequent feelings of guilt as well [10]. In general, neuroticism is characterized by emotional instability, fear, nervous disposition, insecurity, temperamental behavior, impatience, self-consciousness, and impulsive behavior [35,36].

The possible neurobiological bases of neuroticism include the visceral brain (or limbic system), composed by structures such as the medial septum, hippocampus, amygdala, cingulum, and hypothalamus [10,37]. The limbic system comprises a group of structures, including those in the limbic lobe and Papez circuit, which are anatomically interconnected and are probably involved in emotion, learning, and memory [38,39].

There is no doubt about the importance of continuing to study the health implications of neuroticism. Neuroticism has been identified as a strong correlate and predictor of various mental disorders (especially depression and anxiety) and the morbidity and mortality of various physical diseases (i.e., diabetes, cardiovascular disorders, dermatological, respiratory and gastrointestinal problems, fibromyalgia, temporomandibular disorder, chronic pain in general, and even cancer) [9,11,13,40]. Furthermore, the comorbidity among the above reported diseases and neuroticism has been suggested to lead to a greater number of complications, an increase in the frequency of use of health services, worse prognosis, and lesser health-related quality of life [11,12]. Neuroticism has also been considered a risk factor for developing Post-Traumatic Stress Disorder (PTSD) [41,42,43,44] and chronic pain in general (i.e., fibromyalgia, rheumatoid arthritis, etc.) 8 [11,14,40].

Moreover, personality seems to determine the coping strategies that people will use in stressful situations, being those, in turn, that will allow the subject a high or low level of adaptation. The studies that are framed, assuming these statements, evaluate the possible relationship between the levels of a certain dispositional personality variables and the use of certain coping strategies. Specifically, these studies try to determine whether certain variables of personality lead to use coping strategies that at the same time lead to a low level of adaptation to the disease [14]. In this sense, most of the research has focused on neuroticism, and the results show the existence of a significant relationship between high levels of neuroticism and coping strategies that predict poor adaptation to the disease [8,9,13,40]. Thus, empirical evidence has been found that supports an increase in the probability of using ineffective coping strategies for stress management by subjects with high scores in neuroticism, such as, for instance, catastrophizing. High levels of neuroticism might be also be related to pain catastrophizing and a more passive-oriented stress and pain coping strategies [9,45,46,47]. At the same time, passive coping strategies predict higher perceived pain intensity [14,48]. In addition, the relation between neuroticism and chronic pain is mediated by the propensity of high-neuroticism individuals to catastrophize their pain [14]. At this regard, catastrophizing appears to reduce the health-related quality of life and worsen symptoms in several chronic pain conditions, such as fibromyalgia [7,49,50] and migraine [51,52,53]. Therefore, studying neuroticism is useful in order to better understand the pain coping strategies and improve them and the treatment of these patients.

In the same line, it has been reported that the use of maladaptive coping strategies and some personality characteristics such as neuroticism and the presence of personality maladjustments can be considered vulnerability factors for a worse evolution in general and specifically in adaptation disorders. In fact, it seems that the use of these types of coping strategies modulates the relationship between neuroticism and psychological distress [47]. Therefore, they should be considered both in the evaluation and in the development of preventive or intervention strategies, which should be prioritized in people with psychological disorders (i.e., adaptation disorder) that present these characteristics [54].

### 3.2. Migraine and Neuroticism Review’s Findings: Revealing An Important Link

Related to the main objective of this current review, which refers to exploring in depth the relationship between migraine and neuroticism, many affected headache patients show a tendency to experience more negative emotions and stress. This is in line with previous studies, which compare chronic headache patients to healthy controls [55]. Several researchers have studied the relation between migraine and neuroticism based on the Five Factor Model personality approach, which is a hierarchical system of personality in terms of five basic independent domains: neuroticism, extroversion, openness to experience, agreeableness, and conscientiousness. In this frame, neuroticism is conceptualized as the tendency to experience negative emotions, such as anxiety, fear, and frustration [56]. Additionally, some researchers—based on the Minnesota Multiphasic Personality Inventory (MMPI)—have found the “neurotic triad,” which includes hypochondria, hysteria, and depression, in tension-type headache and migraine patients [57]. Furthermore, it has been confirmed that migraine patients have significantly higher levels of neuroticism scores than non-migraine controls, using the Eysenck Personality Questionnaire (EPQ), which assesses neuroticism, extraversion, and psychoticism, together with a Lie/Social Desirability scale [58,59,60].

According to the research evidence, migraine patients usually have a higher level of neuroticism and vulnerability to negative affect, in comparison with non-migraineurs patients [15,16,17,18,19,20,21]. These findings and personality characteristics described are usually associated and are more evident in the cases of greater intensity, duration in time, and frequency of migraine attacks [61]. For that reason, some studies suggest that this type of personality trait is more related to chronic pain than to migraine itself [61,62]; hence, the importance of treating migraines early and properly. Nevertheless, it is a theme still under research and no clear data have been provided at all. What it is evident is that migraineurs studies showed a strong correlation between neuroticism and headache duration [63], though, indeed, when it includes males, no association with duration is found [15]. In the same way, a research comparing migraineurs to their siblings without migraine reported that neuroticism was not associated with attack frequency or severity [64]. Moreover, migraine in childhood diagnosed by physicians and higher trait neuroticism have been significantly related to the prevalence of migraine in adulthood [65]. In fact, neuroticism seems to increase the risk of migraine [66].

A high neuroticism score predisposes to depression and anxiety [16,67], which is clinically interesting, because medication-overuse headache migraine (MOH) patients are diagnosed with depression more frequently than migraineurs [16,68,69].

Previous studies have stated a strong association between high score on the personality domain neuroticism and depression [67]. Although, both migraine and MOH patients have increased risk of developing depression, medication-overuse headache migraine patients have a higher prevalence of depression compared to migraine patients in general [68]. The personality domain neuroticism (risk factor) and extraversion (protective factor) have been linked to general health [70]. A high score on neuroticism has been linked to poor prognosis and health-related quality of life in migraine patients [15,71,72]. A previous study revealed that neuroticism moderated the relationship between depression and migraine [20].

Neuroticism has been also studied in migraine patients compared to tension-type headache (TTH) patients [73]. In this sense, neuroticism and depression scores have been associated with headache frequency (chronic vs. episodic), being higher for migraine and TTH followed by pure TTH [73].

The relation between depression and neuroticism in migraine is an important element. Some authors consider that both neuroticism and depression may influence pain sensitivity and pain perception thresholds in humans [74,75,76,77]. In addition, it is well-known that personality traits’ may affect the individual’s vulnerability and the use of non-adaptive coping strategies facing various types of disease, including pain conditions [50]. In the same line, neuroticism seems to alter pain perception; lower pain thresholds have been found in persons with high levels of neuroticism in comparison with persons with lower levels of neuroticism [78]. Moreover, neuroticism has been related to greater vigilance to pain, pain catastrophizing, and fear of movement/re-injury in patients with non-specific chronic or recurrent pain [75]. Cutaneous allodynia, which may indicate central sensitization, has been shown to be associated with depression in migraineurs [79]. Moreover, depression has been revealed to be associated with an increased risk of transformation from episodic to chronic migraine [80], where neuroticism might be a mediator factor.

In general, migraine patients with comorbid depression and anxiety had more neuroticism than patients without migraines and those with depression or anxiety without migraines [81,82]. As neuroticism is significantly related to symptoms of anxiety and depression [83,84], treatment for migraine might be more effective if it included interventions such as cognitive behavioral therapy (CBT) [85]—which is considered one of the most effective interventions for depression—together with the usual medical treatment [86]. Nevertheless, it is important to be cautious in assuming that depression and neuroticism are related to migraine through the same mechanism. Given that neuroticism is a relatively stable aspect of an individual’s personality, it seems likely that genetically predisposed people will show a certain level of neuroticism throughout their lives, and throughout their lives, at some point, may develop migraine, possibly under the influence of different factors (i.e., hormonal changes and environmental stressors) [66]. By contrast, anxiety and depressive disorders are usually episodic and the mechanism that causes their association with migraine may be different [66].

In fact, it is well-known that personality traits (especially neuroticism) and childhood maltreatment have been independently associated with several negative health outcomes later in life, including migraine [87]. These results are in line with the previous evidence in which personality traits, especially neuroticism, are mediators for the relationship between childhood maltreatment and several mental health variables (i.e., depression, psychological distress, anxiety, substance abuse, alcohol dependence, etc.) [88,89,90,91,92,93,94,95,96]. In this sense, it has been stated that the presence of an overlap in the neural circuitry underlying experiences of physical pain and social pain (e.g., by childhood maltreatment), and understanding social pain as the painful feelings following social rejection or social loss [97]. There is also evidence of neuroticism as a potential mediator for the relationship between childhood abuse and migraine. These results have been confirmed through direct and indirect statistical analyses [87]. The relationship between migraine/headaches and childhood abuse has been established in large non-clinic-based samples together with several small case control studies using clinic and non-clinic-based sampling frames [98,99,100,101,102,103,104,105].

**Figure 3:**
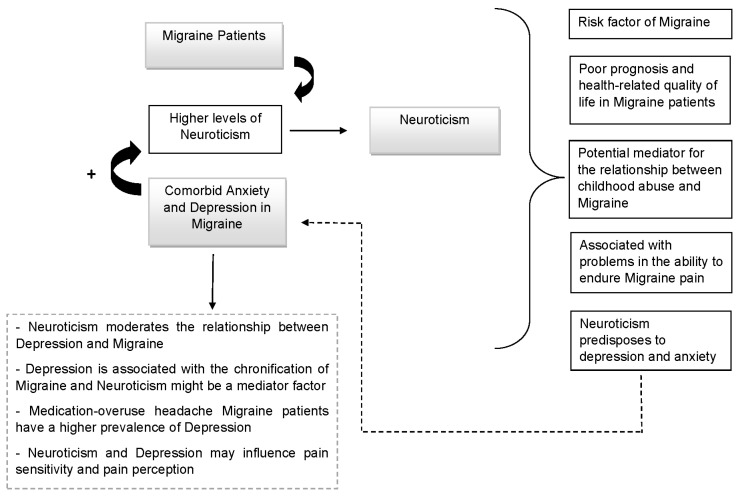
Migraine and Neuroticism.

Lastly, it is relevant to mention that neuroticism seems to be also associated with the ability of old women to endure migraine pain [106]. Some authors propose that the theoretical construct of neuroticism implies that people with a high degree of neuroticism are more sensitive and vulnerable to various stimuli, including pain. Therefore, old women with high levels of stress susceptibility and scoring high in somatic trait anxiety experienced more disability during attacks at low levels of migraine pain intensity, showing a vulnerability to migraine pain [106]. At the same time, it is important to note that ageing is characterized by a reduced ability to cope with challenges [107]. Old women with high levels of certain neuroticism-related personality traits showed difficulties in enduring migraine pain, and possibly a reduced ability to cope with the challenge of migraine pain, including pain of mild intensity [106]. Thus, more research is needed in this regard. The relation between neuroticism and migraine is complex and requires us to studied it more widely. Figure 3 (elaborated by authors) shows the above reported most important elements for understanding the relation between migraine and neuroticism.

## 4. Conclusions

Based on research evidence, migraine patients usually have a higher level of neuroticism and vulnerability to negative affect, in comparison with non-migraineurs and tension-type headache patients. The personality trait of neuroticism has been associated with migraine, although research is needed to clarify potential moderators of this relationship. Migraine patients with comorbid depression and anxiety showed higher levels of neuroticism. At the same time, depression has been related to an increased risk of transformation from episodic to chronic migraine and neuroticism might be a mediator factor on this transformation. Neuroticism might also be a mediator factor in the association between childhood maltreatment and migraine. In addition, neuroticism is considered as a risk factor of migraine and it seems to be associated with problems in the ability to endure migraine pain.

It is worth highlighting the fact that even though migraine is a fundamentally clinical diagnostic disorder whose treatment is relatively simple (outpatient and oral), it is usually underdiagnosed and insufficiently controlled. Chronic migraine can be prevented with proper therapy. Proper treatment of migraine can help reduce the significant loss of productivity that it generates and significantly improve the health-related quality of the lives of people who suffer from it, in the short and long term. It is also recommended that clinicians be aware of the relationship between neuroticism, depression, and migraine and, taking this into consideration, use a multidisciplinary treatment approach in the migraine intervention. Furthermore, the questions about if neuroticism can predict chronification of migraine remains unclear and it is an interesting and necessary future line of research.
